# Selected fruit pomaces: Nutritional profile, health benefits, and applications in functional foods and feeds

**DOI:** 10.1016/j.crfs.2024.100791

**Published:** 2024-06-15

**Authors:** Harsh Kumar, Shivani Guleria, Neetika Kimta, Eugenie Nepovimova, Rajni Dhalaria, Daljeet Singh Dhanjal, Nidhi Sethi, Suliman Y. Alomar, Kamil Kuca

**Affiliations:** a Centre of Advanced Technologies, Faculty of Science, University of Hradec Kralove, Rokitanskeho 62, 50003, Hradec Kralove, Czech Republic; b Department of Biotechnology, TIFAC-Centre of Relevance and Excellence in Agro and Industrial Biotechnology (CORE), Thapar Institute of Engineering and Technology, Patiala, 147001, India; c School of Biological and Environmental Sciences, Shoolini University of Biotechnology and Management Sciences, Solan, 173229, India; d Department of Chemistry, Faculty of Science, University of Hradec Kralove, 50005, Hradec Kralove, Czech Republic; e School of Bioengineering and Biosciences, Lovely Professional University, Phagwara, Punjab, 144411, India; f Department of Pharmaceutical Sciences, Guru Nanak Dev University, Amritsar, 143005, India; g Zoology Department, College of Science, King Saud University, Riyadh, 11451, Saudi Arabia; h Biomedical Research Center, University Hospital of Hradec Kralove, 50005, Hradec Kralove, Czech Republic

**Keywords:** Fruit pomaces, Enrichment, Functional feed, Functional food, Health

## Abstract

The utmost objective of every nation is to achieve zero hunger and ensure the health and well-being of its population. However, in impoverished nations, particularly in rural areas, such issues persist on a daily basis. Currently, there is a growing demand for fruit consumption due to their potential health benefits. Surprisingly, their most prevalent by-product is pomace, which is produced in millions of tonnes and is usually discarded as waste after processing or consumption. Even food produced with these kinds of raw resources can contribute to the objective of eradicating world hunger. Owing to these advantages, scientists have begun evaluating the nutritional content of various fruit pomace varieties as well as the chemical composition in different bioactive constituents, which have significant health benefits and can be used to formulate a variety of food products with notable nutraceutical and functional potential. So, the purpose of this review is to understand the existing familiarity of nutritional and phytochemical composition of selected fruit pomaces, those derived from pineapple, orange, grape, apple, and tomato. Furthermore, this article covers pre-clinical and clinical investigations conducted on the selected fruit pomace extracts and/or powder forms and its incorporation into food products and animal feed. Adding fruit pomaces reduces the glycemic index, increases the fibre content and total polyphenolic contents, and reduces the cooking loss, etc. In animal feeds, incorporating fruit pomaces improves the antioxidant enzyme activities, humoral immune system, and growth performance and reduces methane emission.

## Highlights

• Fruit pomaces are generally considered as waste but are a good source of nutrients.
• Enrichment or development of functional food using fruit pomaces provides enough nutrition to low-income nations.
• Use of fruit pomaces in animal feed lowers the dependency of farmers on high-cost feeds.

## Graphical abstract

**Figure:**
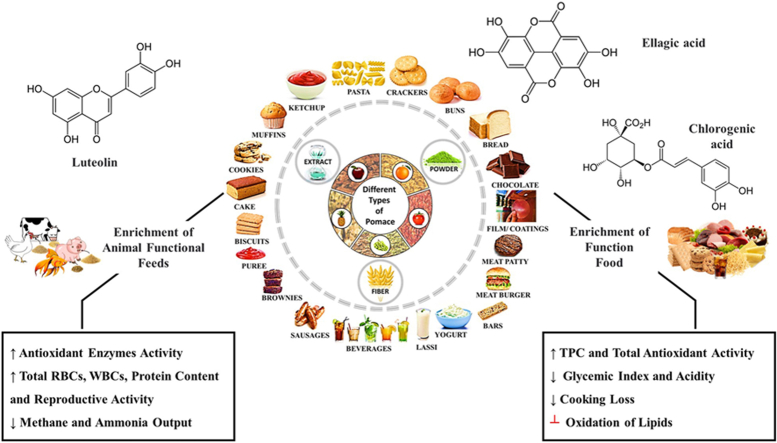

## 1 Introduction

The recent data record as early as August 2023 indicates that a vast population of nearly 238 million people encompassing 48 countries being affected by food crisis, encountering elevation in acute food insecurity levels and impacting every 1 in 5 persons amongst the analyzed population, thus presenting a trend similar to the one witnessed in 2022 (EU, 2023). The current terminology frequently uses “food crisis” while referring to the malnutrition conundrum. In the year 2010, Timmer defined food crisis as “the sharp rise of hunger and malnutrition rates at local, national, or global levels”. The very recent Russo-Ukrainian conflict and COVID-19 pandemic have aggravated the fears of imminent global food crisis (GFC) (Bentley, 2022; Okolie and Ogundeji, 2022). By and large, phenomena threatening food security (FS) such as economic busts, armed conflicts, and climate change are liable to provide impetus for a food crisis (Mavroeidis et al., 2022). Amongst aforementioned reasons, food wastage is also a critical issue contributing to food crisis. Globally, food wastes occur in large quantities and lead to food insecurity in addition to pollution of air (burning), soil (landfills), and water resources (Asif et al., 2023). It has been estimated that nearly one thirds of the total food produced in the world gets wasted due to consumer behaviour, improper handling, and technological issues (Asif et al., 2023).

**Fig. 1:**
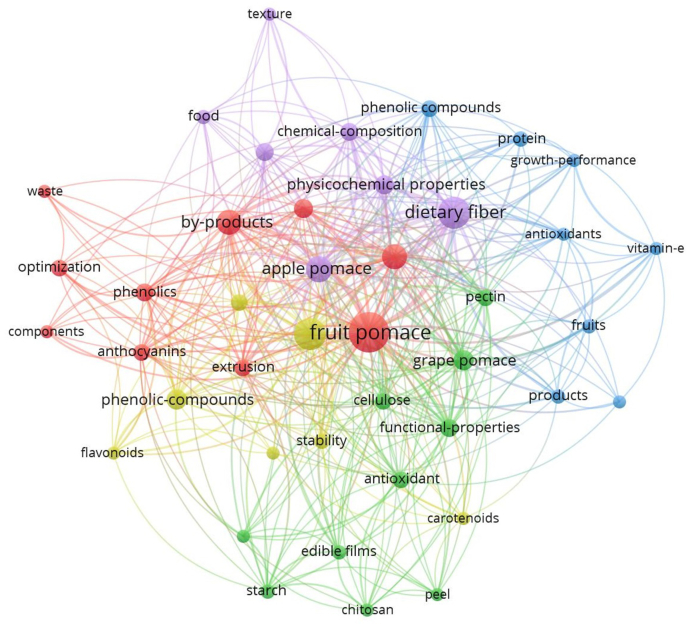
A visual exploration of keyword co-occurrence of fruit pomaces applications.

The annual global food wastage amounts to an estimated ∼1300 million tons as a consequence of the improper storage facilities (1–17 %), processing techniques (0.5–25 %), climatic effect, harvesting technologies (2–20 %), and customer behaviour (1–30 %) (Awasthi et al., 2020; Khan et al., 2022). Amongst various sources of food waste, vegetable and fruit processing waste constitute the biggest share (∼50 %) (Muniz et al., 2020). The vegetable and fruit processing wastes are mainly composed of seeds, peels, and pomace. These waste products are usually disposed of without any processing or recycling, thus causing environmental pollution (Razzaq et al., 2020). However, many researchers have conducted projects on food wastes and have displayed that these are an abundant source of minerals, vitamins, dietary fibre, and bioactive compounds (Saini et al., 2019; Iqbal et al., 2021). Fruit pomaces are the byproducts obtained by pressing or crushing of entire fruits to extract their juice, particularly in fruit processing companies and wineries (Erinle and Adewole, 2022). They rapidly undergo oxidation and fermentation reactions in the presence of oxidants, heat and light, leading to the degradation of important compounds contained within them (Erinle and Adewole, 2022). Their disposal creates an environmental health hazard because to their huge volume and moisture content, making them an ideal breeding ground for harmful microorganisms to flourish (Erinle and Adewole, 2022). Data suggests about 4 million tons of apple pomace being produced globally every year (Gołębiewska et al., 2022). The discarded pomace could further undergo fermentation, thereby posing a potential threat to the environment. The United States of America (USA) expenditure on apple pomace disposal accounts to $10 million per year (Awasthi et al., 2021). A 2008 study by Broome and Warner (2008) has stated that the winery industry wastes were composed of around 45 % grape pomace (Ilyas et al., 2021). Citrus fruits are processed to extract citrus juices that also generate wastes that constitute approximately 45–60 % of fruit weight (Fernández‐López et al., 2009). Similarly, pineapple pomace attributes to nearly 30 % of the fruit pulp with a juicing recovery assumption up to 70 % (Banerjee et al., 2018). Lu et al. (2019) have reviewed tomato pomace yields across the world and were estimated to be 5.4–9.0 × 10^6^ tonnes. Consequently, the commercial use of various fruit pomaces has become a popular research interest. Currently, it is used as a food ingredient substitute, feed ingredient, and nutritional supplement for animal and human consumptions (Acharjee et al., 2021; Ali et al., 2021; Ao et al., 2022; Kesbiç et al., 2022; Rehal et al., 2022; Gumul et al., 2023; Haščík et al., 2023). With the increase in application of fruit pomace as evident from the Web of Science (WOS) data between years 2001–2024 and hence, has also included fruit pomace as the search string. Nearly 157 peer-reviewed research and review articles have been published on fruit pomaces, along with their complete records, citations, and saved as plain text format. The VOSviewer (version 1.6.19) software tool has further been utilized for mapping down and inspecting the keyword, co-occurrence analysis. Additionally, Fig. 1 demonstrates the co-occurrence analysis keyword that describes functional properties, dietary fibre, phenolic compounds, extrusion, food, waste, and by-products as potential areas linking to fruit pomaces. However, Granato and his team (2020) have reported a scientific definition for functional foods as “either natural or industrially processed foods that when consumed regularly as part of a diverse diet at effective levels are believed to offer health benefits beyond their fundamental nutritional value”.

**Fig. 2:**
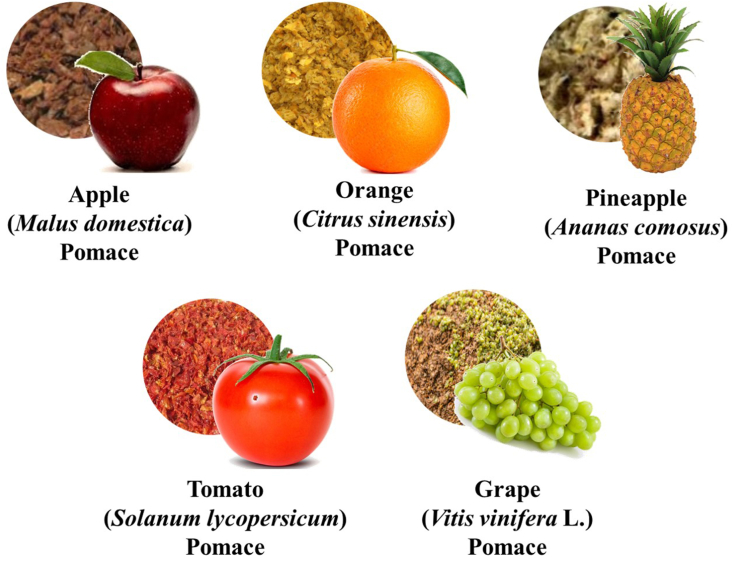
Different types of commonly available fruit pomaces.

The Sustainable Development Goals (SDGs) by United Nations (UN) recommends food waste management to attain its objectives: SDG 2 (zero hunger) and SDG 3 (ensure healthy lives and well-being) (UN, 2024). Therefore, the current review is an endeavour to provide phytochemical as well as nutritional profiling information on selected fruit pomaces, such as apple (*Malus domestica*), orange (*Citrus sinensis*), grape (*Vitis vinifera*), tomato (*Solanum lycopersicum* L.), and pineapple (*Ananas comosus*) (Fig. 2), and hammer out the pre-clinical, and clinical studies data. Furthermore, this review also highlights the use of above-mentioned fruit pomaces in enrichment of different animal feeds and food products.

## 2 Nutritional and phytochemical profile of selected fruit pomaces

### 2.1 Proximate composition

Table 1 illustrates nutritional composition of selected fruit pomaces along with their moisture, protein, ash, carbohydrate, and fibre contents. The carbohydrate content in apples is nearly 14% of the total nutrient composition (Skinner et al., 2018a, Skinner et al., 2018b). Also, the carbohydrate content in apple pomace is greater than apples (Table 1). The sucrose concentration in apple pomace is variable because of its high variation amongst apple cultivars (Queji et al., 2010). It has been reported that fructose and glucose constitute a large proportion of the total carbohydrates in both apples as well as apple pomace (Bhushan et al., 2008). On comparison between the apples and apple pomace for fructose and glucose content, it has been observed to be higher in pomace, probably due to the presence of the seeds which contain sugar in addition (Skinner et al., 2018a, Skinner et al., 2018b). The grape pomace contains high fibre content ranging between 26 % and 70 % and having exceptional lignin levels ranging from 18 % to 55 % (Ilyas et al., 2021). The sugar content comparison between red and white wine shows a lower content varying between 4 % and 9 % in case of red wine pomace as compared to 28 %–31 % in case of white wine pomace (Ilyas et al., 2021). Figuerola et al. (2005) have studied the composition of orange pomace cultivar ‘‘Valencia’’ based upon its dry matter and found out lesser total dietary fibre of 64.3 %, and lesser ash of 2.71 %, but comparable protein content of 6.70 %. The differences in composition could be attributed to differences in growing conditions and fruit maturity time (Sturm et al., 2003). The proximate composition of pineapple pomace suggests fibre (45.22 ± 3.62 %) as the major component, with 98.28 % of it being composed of the insoluble fraction (Selani et al., 2014). These findings are in concurrence with the reports by Huang and his team (2011), which documents total dietary fibre to be 42.2 % in pineapple peels, wherein the insoluble fraction forms the main constituent (86.02 %). Selani and his researchers (2014) conducted studies on pineapple pomace and reported similar carbohydrates content (as described in Table 1) as reported by Huang et al. (2011), who document 42.3 % content in pineapple peel.

**Table 1:** Proximate composition in selected fruit pomaces.

Apple (%) on fresh weight basis	Grape (%) on dry weight basis	Orange (%) on fresh weight basis	Pineapple (%) on dry weight basis	Tomato (%) on dry weight basis	Reference
Moisture (66.4–78.2); Ash (0.38–1.6); Proteins (4); Carbohydrates (9.5–22); Fibre (9)	Moisture (3.33); Ash (4.6); Proteins (8.49); Carbohydrates (29.2); Fibre (51.3)	Moisture (10.5); Ash (3.6); Proteins (6); Carbohydrates (77.7); Fibre (40.4)	Moisture (3.7); Ash (2.2); Proteins (4.7); Carbohydrates (43.4); Fibre (45.2)	Moisture (5); Ash (3.5); Proteins (32.6); Carbohydrates (43.3); Fibre (29.4)	Selani et al. (2014); O’Shea et al. (2015); Beres et al. (2016); Özbek et al. (2020); Spinei and Oroian (2021); Costa et al. (2022); Kauser et al. (2024)

Del Valle and his research groups (2006) have found that non-dried tomato pomaces contain very high moisture (64.31–92.55 %) content in plant samples collected at different production stages. A similar study by Bhat and Ahsan (2018) conducted on tomato pomace states moisture content of 87.63 % in fresh tomato pomace, 7.37 % content in freeze-dried pomace, and 4.77 % content in cabinet-dried ones. The presence of large amounts of moisture in non-dried tomato pomace makes them highly susceptible to undesirable fermentation and microbial spoilage. And hence, their long-term storage is not feasible. Therefore, pre-treatment of food wastes with high moisture levels through drying could help extend their stability.

### 2.2 Amino acids

Amino acids, by definition are essential biomolecules that acquire tremendous significance in tissue protein building and human health. These are documented to play significant role in diseases like infertility, neurological dysfunction, and intestinal disorders, with positive outcomes and could also be utilized as fingerprints for uncovering the varietal origin of fruits (Botoran et al., 2019; Turkiewicz et al., 2020). Another crucial factor driving the apple aroma during production is the harvesting time, with earlier harvesting causing a decrease in the aroma spectrum (Radenkovs et al., 2018). Amino acids are regarded as the precursors of aroma during fruit maturation time and are therefore used for the aroma component synthesis. Hence, these become the second most significant source of developing volatile aroma compounds in plants (Espino-Díaz et al., 2016; Ito et al., 2017). Wicklund and his team (2021) have stated that amino acid accumulation (glutamic and aspartic acid in this case) draws direct correlation with horticulture conditions, especially nitrogen presence. On a broader spectrum, primarily cultivar and secondly the year of cropping are the major contributors towards fruit composition in apple, considering both primary as well as secondary metabolites (Bourvellec et al., 2015). Chikwanha et al. (2018) have investigated three grape varieties (Shiraz, Pinotage, and Sauvignon Blanc) and reported amino acid presence in them, influenced by the type of variety and drying methods. Researchers have explored pineapple processing waste (PPW) such as crown, peel, pomace, and core, and found amino acids in higher number (10 amino acids) in pomace, of which 52.46 % were essential and 47.54 % were non-essential amino acids (Sengar et al., 2022). On further investigation of the solid pomace residue obtained after pineapple juice extraction, essential amino acids such as histidine, lysine, leucine, methionine, phenylalanine and isoleucine (Table 2) and non-essential amino acids such as proline, alanine, tyrosine, and glutamic acid were found (Sengar et al., 2022). Tomato pomace is also a rich source of amino acids such as aspartic acid, glutamic acid, arginine, alanine, leucine, isoleucine, and glycine (Table 2) (Chabi et al., 2024).

**Table 2:** Availability of amino acids in selected fruit pomaces.

Apple (mg/100g)	Grape (mg/g)	Orange	Pineapple (%)	Tomato (mg/100g)	Reference
Alanine (5.6), glycine (12.5), threonine (0.7), serine (1.5), valine (2.2), leucine (0.5), isoleucine (1.1), methionine (1.9), proline (0.06), aspartic acid (63.5), phenylalanine (0.6), lysine (0.2), glutamic acid (2.5)	Alanine (4.2), glycine (8), threonine (41.1), serine (4.5), valine (4.2), leucine (6.2), isoleucine (3.5), methionine (0.3), proline (7.8), aspartic acid (7.4), phenylalanine (5.8), lysine (3.2), glutamic acid (12.8), arginine (4.2), asparagine (1.7), cysteine (0.1), glutamine (20.8), histidine (3.1), tyrosine (4.4)	NR	Alanine (0.5), leucine (0.8), isoleucine (0.01), methionine (5.6), proline (42), phenylalanine (0.7), lysine (1.1), glutamic acid (0.1), tyrosine (4.8), histidine (44.1)	Alanine (710), glycine (630), threonine (550), serine (170), leucine (1070), isoleucine (690), methionine (270), aspartic acid (1570), phenylalanine (610), lysine (880), glutamic acid (7210), histidine (260), arginine (1460), cysteine (230), tyrosine (690)	Chikwanha et al. (2018); Fărcaș et al. (2022); Sengar et al. (2022); Chabi et al. (2024)

NR, not reported.

### 2.3 Minerals

The American diet contains potassium and calcium as micronutrients of concern. In USA, the richest calcium source amongst commonly consumed fruits is oranges (Slavin and Lloyd, 2012). Table 3 also illustrates the highest calcium content in orange pomace. Apple pomace has the highest iron content in comparison to the remaining minerals (Table 3). Ahmed and his team (2020) have studied 10 diverse grape pomace (GP) varieties (Alfonse, Büzgülü, Ekşikara, Antepkarası, Çalkarası, Dimlit, Marcaş, Redglob, Topacık, Hönüsü) from Turkey region. The results revealed that iron, phosphorus, potassium, and zinc were the major minerals found invariably in the varieties. Detailed insights into the mineral content of pineapple fruit showed relatively higher content in the exocarp or peel than in the mesocarp or pomace and endocarp or core of the fruit, attributed to leaching out of most minerals during juice extraction process and hence, lower mineral content in the mesocarp or pomace (Sengar et al., 2022). It has been observed that in tomatoes, the farming conditions and level of ripening influences the mineral composition (Caruso et al., 2019; Ramesh et al., 2021). Also, tomato pomace along with seeds and peels are valuable and abundant source of a variety of minerals *viz*, potassium, phosphorus (not present in pomace without peels and seeds), calcium, iron, magnesium, and sodium (Chabi et al., 2024).

**Table 3:** Minerals in selected fruit pomaces.

Apple (%) on dry weight basis	Grape on dry weight basis	Orange (mg/100 ml) on fresh weight basis	Pineapple (g/kg) on dry weight basis	Tomato (μg/g) on dry weight basis	Reference
Calcium (0.06–01), phosphorous (0.07–0.076), magnesium (0.02–0.36), iron (31.8–38.8 mg/kg)	Calcium (9.9 mg/kg), phosphorous (2.7 mg/kg), magnesium (0.8 g/kg), potassium (13.90 g/kg), sodium (0.2 g/kg), sulphur (1.5 g/kg), manganese (13 mg/kg), zinc (25 mg/kg), copper (49 mg/kg), iron (361 mg/kg), selenium (0.2 mg/kg), cobalt (0.4 mg/kg)	Calcium (411.2), potassium (411.2), magnesium (57), zinc (0.2), iron (0.9)	Calcium (0.3), magnesium (0.8), potassium (9.8), phosphorus (0.3)	Calcium (146.4), potassium (2686.9), iron (29.2), magnesium (281.3), sodium (210.3)	O’Shea et al. (2015); Spinei and Oroian (2021); Sengar et al. (2022); Chabi et al. (2024); Kauser et al. (2024)

### 2.4 Vitamins

Apart from minerals, apple pomace is also abundant source of vitamin C and A (Bhushan et al., 2008). A study by Spinei and Oroian (2021) documented that dried grape pomace contains vitamin E and vitamin C in 5 mg/kg and 26.2 mg/g weight respectively. Another study by Afrin et al. (2022) reported that variation in drying temperature for orange pomace could influence the vitamin C content, for example at 50 °C, 60 °C, and 70 °C the quantity of vitamin C was recorded to be 119.36 ± 0.91 mg/100g, 114.42 ± 1.07 mg/100g, and 110.23 ± 1.09 mg/100g respectively. The tomato pomace also contains vitamin B_12_ and vitamin C in 1110 mg/100g and 14 mg/100g composition (Chabi et al., 2024).

### 2.5 Phytochemicals

Phytochemicals derive their name from “phyto”, a Greek word which means "plant". These are the biochemicals produced through primary and/or secondary metabolic processes in the plants. The phytochemicals not only possess diverse biological activities but also are essential in plant growth as well as defence against predators or pathogens. Though phytochemicals are usually not regarded as essential dietary components and necessary for sustenance of normal life, but they have been documented to possess certain pharmacological properties (Sharma et al., 2019). A number of epidemiological studies have suggested that phytochemical rich diets not only protect humans against chronic disorders but also help protect the cellular mechanisms from oxidative damage (Sharma et al., 2019). Recently, the phytochemical applications have also been extended to other areas such as functional foods and nutraceuticals. Table 4 demonstrates different types of phytochemical compounds especially polyphenols that are found in selected fruit pomaces.

**Table 4:** Polyphenols in selected fruit pomaces.

Class	Apple	Grape	Orange	Pineapple	Tomato	Reference
Flavonoids	Catechin, epicatechin, quercetin, procyanidin B_2_	Quercetin, catechin, procyanidin B_2_	Eriocitrin, narirutin, naringenin, apigenin, apigenin 7-*O*-glucoside, tangeretin, diosmin, luteolin, luteolin 7-*O*-glucoside, quercetin, quercetin 3-*O*-rhamnoside, kaempferol 3- *O*-rutinoside, rutin, quercetin 3,4′-diglucoside, hesperidin, hesperidin 7-*O*-glucoside	Epicatechin	Epicatechin, quercetin, rutin	Sepúlveda et al. (2018); Multari et al. (2020); Spinei and Oroian (2021); Ershidat (203); Kauser et al. (2024)
Phenolic acids	Chlorogenic acid, cinnamic acid, caffeic acid	Gallic acid	*p*-hydroxybenzoic acid, *p*-coumaric acid, ferulic acid, caffeic acid, chlorogenic acid, neochlorogenic acid, vanillic acid	Gallic acid, chlorogenic acid, caffeic acid, *p*-coumaric acid, cinnamic acid	Coumaric acid, caffeic acid, chlorogenic acid, gallic acid, syringic acid, vanillic acid, trans- cinnamic acid, ellagic acid, ferulic acid	Montalvo-González et al. (2018); Multari et al. (2020); Spinei and Oroian (2021); Ershidat (203); Kauser et al. (2024)
Tannins	NR	Hydrolysable tannins, catehic tannins	NR	NR	NR	Spinei and Oroian (2021)

NR, not reported.

Li and his team (2020) have analyzed the polyphenols obtained from apple pomace through different extraction techniques and hydrolysis procedures. The results from aqueous-methanol extract revealed free polyphenolic compounds presence, with 5.56 mg of gallic acid equivalents (GAE)/g the dry weight (DW) of pomace. The major compounds were identified as quercetin-3-O-galactoside, chlorogenic acid, phloridzin, and quercetin-3-O-rhamnoside. Use of diverse sequential bases followed by acid hydrolysis and extraction by solvent system, diethyl ether/ethyl acetate on the residue yielded bound polyphenols in 6.82–8.12 mg GAE/g the dry weight. On subjecting the aqueous-methanolic residue to base hydrolysis, the major phenolics extracted include phloridzin, quercetin-3-O-galactoside, and 4-hydroxybenzoic acid. Subsequent acid hydrolysis of the residue further released considerable amounts of isoferulic acid and 4-hydroxybenzoic acid. Chamorro and his research group (2012) on investigation found out that grape pomace treated with tannase enzyme for 24 h converted the galloylated form to free form of catechin, thus, releasing gallic acid as well as enhancing the antioxidant activity. Another study by Drosou et al. (2015) on air dried red grape pomace of Greek variety Agiorgitiko found out the phenolic content to be 438984 ± 4034 ppm GAE. The authors used ultrasound assisted extraction (UAE) technique with water: ethanol (1:1) for extraction of phenolic compounds. The air-dried pomace extracts contained very high total flavan-3-ol content of 43469 ± 1210 ppm CE (catechin), total flavonol content of 4484 ± 108 ppm QE (quercetin), and total anthocyanin content of 34188 ± 362 ppm Mv-3-glc eq (malvidin -3-O-glucoside equivalents). Multari et al. (2020) have investigated the phenolic compounds present in orange pomace (OrP) through Ultra-performance liquid chromatography-mass spectrometry (UPLC-MS/MS) analysis and found out the hesperidin quantity of 377 ± 17.2 mg/kg. Narirutin concentration in OrP (141 ± 11.5 mg/kg) was nearly ten times greater than that in orange juice (15.6 ± 2.89 mg/kg). Luteolin concentration in OrP was found out to be 41.7 ± 3.13 mg/kg. The phenolic acids, *p*-coumaric acid (<0.5 mg/kg) and ferulic acid (>5 mg/kg) levels in OrP were comparable to the levels reported by Montero-Calderon and his team (2019). Sepúlveda et al. (2018) evaluated pineapple waste for extracting polyphenols and glycosides using autohydrolysis, an alternative technique that utilizes only water as solvent for the extraction process. Box-Behnken design (BBD) experiments were conducted using variable temperatures (between 150 and 200 °C), reaction time (between 15 and 45 min), and solid-liquid ratio (ranging from 1:40–1:10 w/v). Their findings have revealed that the extracted total polyphenol content (1.75 g/L) was highest at 200 °C temperature, 1:10 w/v i.e., solid-liquid ratio, and 30 min time. Among all treatment groups gallic acid, chlorogenic acid, hydroxybenzoic acid, epicatechin, caffeic acid, and coumaric acid were detected. Ershidat (2023) have investigated tomato pomace and found that blending frozen (BF) method was best for extracting various components *viz*, total phenol content of 1297.4 μg GAE/g and total flavonoid content of 462.5 μg QE/g. It was also observed that ellagic acid and gallic acid were predominant amongst the phenolic compounds present in tomato pomace. Another study by Vorobyova and team (2022) on tomato pomace examined the deep eutectic solvents (DESs) efficacy for extraction of polyphenolic compounds with the aid of ultrasound. The most abundant components in these extracts were detected to be flavanols and phenolic acids. The phenolic compound, chlorogenic acid was found out to be the major component in tomato extracts (Vorobyova et al., 2022).

## 3 Pre-clinical studies of selected fruit pomaces

Cho et al. (2013) conducted a study on apple juice concentrate (AC) and apple pomace (AP) supplementation influencing fat loss, lipid metabolism and body weight in obese rats being fed on a high-fat diet. Obese rats on high-fat diet (HFD) were divided into three groups with eight animals in each: HFD control, HFD with 10 % (w/w) AC and HFD with 10 % (w/w) AP. For better understanding of the diet, another group (n = 8) of rats fed on normal diet was also assigned. The study extended for a period of 5 weeks and the investigations revealed lowering of body weight gain, serum total cholesterol, triglyceride concentrations, low-density lipoprotein, white adipose tissue (WAT) weight, lesion scores and epididymal adipocyte size and significant increase in brown adipose tissue weight and serum high-density lipoprotein concentration in both the AC and AP groups in comparison to the HFD group. Additionally, the atherogenic indices were significantly lower in the AC and AP groups compared to the HFD group.

A study by Skinner and team (2018) was carried out for replacing calories with apple pomace in Western or standard diets to establish their impact on genetic regulation of hepatic lipid metabolism along with risk assessment of non-alcoholic fatty liver disease (NAFLD). Random grouping (n = 8 rats/group) of female Sprague-Dawley rats was done and fed on isocaloric diets as described by American Institute of Nutrition (AIN); AIN-93G/10 % w/w of apple pomace (AIN/AP) and AIN-93G. Other assigned groups included female rats fed on isocaloric diets; Western (33 % sucrose and 45 % fat) and Western/10 % w/w of apple pomace (Western/AP) for eight weeks. The study results revealed that fibre-rich apple pomace diet i.e., Western/AP attenuated the fat vacuole infiltration, increased monounsaturated fatty acid concentration, and hepatic triglyceride storage due to expression of hepatic diacylglycerol O-acyltransferase 2 (DGAT2) gene and higher circulation of bile induced by a Western diet. The authors further investigated the antioxidant-rich apple pomace, a “waste” byproduct for its potential as functional food that could alleviate non-alcoholic steatohepatitis (NASH). To establish correlation between NASH and apple pomace, growing Sprague-Dawley rats (female) were made to consume any one of the henceforth mentioned four diets for a period of 8 weeks: AIN-93G diet, AIN-93G/10 % g/kg of apple pomace (AIN/AP) diet, Western/10 % of apple pomace (Western/AP) diet or Western diet. Substitution with apple pomace in diet decreased the Western-diet induced histological inflammation in liver and had positive influence on adipose tissue and liver monounsaturated and saturated fatty acid content. Further results imply that rats fed on Western/AP displayed downregulation of gene expression in adipose and hepatic proinflammatory cytokines and improvement of antioxidant status on comparison to Western diet fed rats.

Another study explored the effects of supplementing grape pomace (GP) and its extract (GPE) in Wistar rat diet on liver, muscle, and adipose tissues that are insulin sensitive, was conducted using rat model for metabolic syndrome (MetS) (Lanzi et al., 2016). A diet containing high-fat-fructose (HFF) was given to the rats for development of MetS. Results have shown that GP supplementation of 1 g/kg/day helped prevent systolic blood pressure (SBP), increase c-reactive protein (CRP) and triglycerides and partially reduced insulin resistance whereas GPE supplementation of 300 mg/kg/day led to partial attenuation of triglycerides and SBP and significant prevention of inflammation and insulin resistance.

Yu and his team (2017) have analyzed the GP supplemented diet impacting the blood lipid profile, expression of hepatic genes related to lipid metabolism, and body weight with the assistance of young rat model. The study included twenty, 7 weeks old Sprague-Dawley rats (female) divided randomly into 4 groups, and fed with variable amounts of GP i.e., modified AIN-93G diets having 0 % GP (control), 6.9 % GP, 13.8 % GP, and 20.7 % GP for a period of 10 weeks. The GP-containing diet had no influence on the rat body weight. The increase in GP content contributed to decrease (p < 0.05) in very low-density lipoprotein (VLDL) and blood triglycerides, slight but statistically insignificant increase in high density lipoprotein (HDL), significant increase (p < 0.05) in low-density lipoprotein (LDL) and total cholesterol (TC), decrease in blood glucose levels, and slight increase in alanine transaminase (ALT) level. The hepatic gene expression associated to lipid metabolism/hydrolysis and fatty acid synthesis was moderately downregulated with GP diet. This study concluded that a routine intake of diet consisting appropriate quantities of GP could help prevent obesity and reduce body fat accumulation.

Owoeye and Farombi (2015) have investigated effect of tomato pomace powder (TPP) on mercuric chloride (HgCl_2_) intoxicated rats. Thirty rats (male) were taken and randomly assigned five groups of 6 rats per group: Control; propylene glycol; HgCl_2_ (4 mg/body weight) from 5th to 19th day; TPP (50 mg/kg body weight) for 19 days; TPP + HgCl_2_ combination; HgCl_2_ (4 mg/body weight) from 5th to 19th day of the experiment + TPP (dose of 50 mg/kg body weight) for 19 days. All treatment groups were administered orally by gavage. Treating rats with TPP before administering HgCl_2_ led to significant reduction in the HgCl_2_ effect on various parameters that could be ascribed partly to the antioxidant potential of TPP. TPP provided protection against HgCl_2_-induced altered motor anomaly as well as microanatomy of rats’ hippocampus, cerebral cortex, and cerebellum. Espinosa-Juárez and co-workers (2017) have investigated Saladette tomato pomace (STP) lipidic extract effect on the bladder and prostate in a high-carbohydrates diet induced obesity rat model. Rats fed on high-sucrose exhibited higher prostate weight, epithelial and stromal hyperplasia, and increased contractility in prostate. Treatment of rats with STP improved prostate contractility, diminished prostate hypertrophy and hyperplasia in the obesity model. Another study was conducted by Fouda et al. (2024) to examine tomato pomace (TP) for the presence of antioxidants, and its capability to provide protection against indomethacin triggered ulceration and erosion. Two sets of microcapsules were formulated; one with tomato pomace extract (TPE) alone and other in combination with probiotics to further enhance its potential effect. The encapsulated forms were investigated for their efficacy in drug (indomethacin)-treated rats. The extracts demonstrated antioxidant potential along with high levels of polyphenols and carotenoids (15 mg/g extract). Both the formulations of TPE microcapsules (alone and with probiotics combination), demonstrated a protective action against enterocolitis by reduction of inflammation and oxidative stress, as indicated by the decreased intestinal and stomach malondialdehyde (MDA), interleukin 6 (IL-6), interleukin 1 beta (IL-1β), tumour necrosis factor alpha (TNF-α), and nitric oxide (NO) levels and increased superoxide dismutase (SOD), catalase (CAT), and reduced glutathione (GSH) levels. The produced microcapsules could therefore be proposed as promising candidates, acting as shield against gastric erosion and ulcers.

## 4 Clinical studies of selected fruit pomaces

A study by Guzman and his research group (2020) established the impact of apple pomace (AP) addition to pure apple juice (AJ, 100 %) especially on postprandial blood glucose response in comparison to whole apple fruit (WAF) or equivalent sugar containing apple juice when given to healthy human subjects. The findings showed that addition of 5 g fibre from apple pomace into 235 g of juice did not diminish the maximal postprandial glycemic concentration. However, the time to arrive at maximal insulin and glucose concentration was significantly delayed in trial group that received pomace fibre along with apple juice in contrast to WAF or apple juice alone. Trimethylamine N-oxide (TMAO), an amine oxide is regarded as a novel marker for increased risk associated with cardiovascular diseases. Annunziata et al. (2019) have assessed TMAO-reducing effect in humans by testing a novel nutraceutical formulation (Taurisolo®) prepared from grape pomace extract. To examine the effectiveness of Taurisolo®, randomized, placebo-controlled cross-over clinical trial was conducted. In acute cases, the maximum resveratrol (RSV) levels were detected in whole blood as well as serum, after 60 min of Taurisolo® administration. In chronic cases, a significant RSV increase was noticed in serum after four-week treatment. On comparing the treatment group (63.6 %) to placebo group (0.54 %), the TMAO levels were significantly (p < 0.0001) decreased after 4 weeks. The study concluded that Taurisolo® could represent a useful and novel natural remedy for reducing prognostic markers in incident cardiovascular diseases (CVD). Martínez-Maqueda and team (2018) endeavoured at evaluation of grape pomace effect on markers of MetS as GP is abundant in extractable as well as non-extractable polyphenols. Fifty subjects, out of whom 22 were women, ranging between age 20–65 and having at least two metabolic syndrome factors were assigned randomly to dried GP (daily dose 8 g) group or the control group. The study was a crossover design that lasted for 6-weeks and a wash out of 4-week. Sample collection was done at the time of beginning as well as at the finish of both periods; wherein 50 % subjects underwent a glucose tolerance test (oral) both at the very beginning and at the ending of the GP supplementation period. It was observed that GP supplementation led to significant improvement in fasting insulinaemia (p < 0.01), with no effect on cardiometabolic risk parameters. Urquiaga and team (2015) have studied that red wine grape pomace flour (WGPF) made from Cabernet Sauvignon variety of red wine grapes, on intake diminished MetS in humans. For evaluation of WGPF impact on MetS components, a 16-week long longitudinal intervention study was designed. 38 males of age group 30–65 years, having minimum of one MetS component, were assigned randomly to the control (n = 13) or the intervention group (n = 25). The intervention group received 20 g WGPF per day at lunch. The WGPF meal constituted 822 mg polyphenols, 10 g dietary fibre, and exhibited antioxidant activity (7258 ORAC units). Both the groups were instructed to follow their regular lifestyles and eating habits. Clinical evaluation, biochemical blood analyses, and anthropometric measurements were done both at the beginning as well as at the end of study. The results revealed that WGPF intake caused significant decrease in systolic as well as diastolic blood pressure and fasting glucose levels. Plasma δ-tocopherol and γ-tocopherol concentration was enhanced and the plasma protein carbonyl groups were also diminished significantly in WGPT group. However, there was insignificant effect on the waist circumference, triglycerides, HDL cholesterol, vitamin C, and total antioxidant capacity not just in groups but also between groups. This study concluded that WGPF diet (rich in polyphenol antioxidants and fibre) when consumed as food supplement with regular diet helps improve glycaemia, postprandial insulin, and blood pressure.

Dennis-Wall and his team (2019) analyzed citrus pomace for its effect on frequency of stool in healthy adults. The trial was randomized, blinded and controlled, wherein healthy adults (consisting 62 % females) were given orange pomace containing beverage (n = 111, 473 mL/day, with 10 g fibre/day) or control (n = 110) for a duration of 3 weeks. Self-assessment scores were determined through stool frequency, Gastrointestinal Symptom Rating Scale (GSRS), and Bristol Stool Form Scale (BSFS). Stool microbiome was analyzed using quantitative polymerase chain reaction (qPCR) and 16S rRNA sequencing. Results revealed that the mean total daily intake of fibre was higher in case of pomace than control (p < 0.0001). Also, mean weekly frequency of stool was higher, thus indicating improved laxation in case of pomace than control (p = 0.0281) and increase from baseline was observed with pomace (p = 0.0003) whereas it remained unchanged with control. A similar trend was observed in the mean BSFS scores that were higher in case of pomace than control (p = 0.04). However, the GSRS syndrome scores, especially gas and bloating symptoms, were higher with citrus pomace, thereby suggesting fermentation. Pomace intake aided increase in operational taxonomic units (OTUs) in faeces, corresponding to *Ruminococcaceae* and *Lachnospiraceae*. Chen and his research group (2017) gave a hypothesis on orange pomace (OP) that it has diminishing effect on postprandial glycemic levels in response to high fat/carbohydrate breakfast and lunch. The researchers conducted an acute clinical trial that was randomized, placebo-controlled, double blind, crossover including 34 overweight male participants who consumed different combination (255 g placebo (PLA) or 35 % OP, low orange pomace (LOP) or 77 % OP high orange pomace (HOP)) dosage of OP beverage in breakfast. The blood samples at various intervals of 0 min, 10 min, 20 min, 30 min, 45 min, 1 h, 1.5 h, 2 h, 3 h, 4 h, 5 h, 5.5 h, 6 h, 6.5 h, 7 h, and 8 h were collected. The consecutive meal i.e., the lunch was given after 5.5 h of blood withdrawal. The findings displayed that OP supplementation reduced the post-breakfast insulin levels by ≥ 10 % and the LOP delayed the maximal response by 14 min in contrast to that of PLA (46 min, p ≤ 0.05). The final outcome of the study suggests that OP assists in diminishing postprandial glycemic responses against a high fat/carbohydrate breakfast and lunch in overweight men. Dong et al. (2016) have investigated the satiation of orange juice (OJ) when orange pomace fibre (OPF) of 5.5 g was added, compared to whole orange (WO) preparation made by chopping and blending it into a puree/liquid, through a randomized, controlled, double blind trial. The results revealed that addition of OPF (5.5 g) to OJ or other orange-flavoured beverage brought significant increase in composite satiety scores in comparison to OJ or control groups (p < 0.0001). Also, the effect on satiety was comparable to WO. The research concluded that OPF addition to OJ was effective in increasing the satiety to the same extent as WO compared to OJ.

## 5 Applications of selected fruit pomaces in food enrichment

**Fig. 3:**
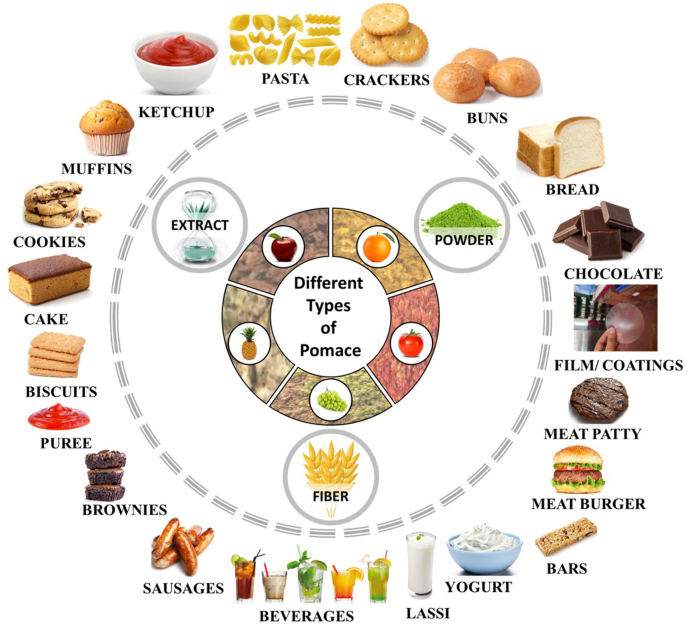
Selected fruit pomaces fractions and their utilization in functional foods development.

The by-products generally represent the total vegetable or fruit residue post pressing. The pomaces can be characterized as cell wall components which are composed of fibrous substances such as hemicelluloses, cellulose, and water-soluble pectin and exhibit good functional properties *viz*., water holding, gelling, and binding abilities (O’Shea et al., 2015). Thus, suggesting that apart from possessing good nutritional properties, these may also play important role in food formulations. Recent trends suggest utilization of fruit pomaces in various forms of extracts, fibre, and powder etc., in diverse food formulations and development of a wide array of food products such as muffins, cookies, pasta, cakes, biscuits, brownies, breads, buns, crackers, chocolates, bars, ketchup, purees, ready to eat munchies, yogurt, beverages, meat-based patties, sausages, burgers, coatings and films (Fig. 3), that have been comprehensively discussed in the forthcoming subsections.

### 5.1 Bakery-based foods

Bakery-based foods such as bread, cookies, biscuits, cakes, crackers, brownies, buns, pasta, and muffins are essential component of a regular diet (Fig. 3). These foods offer substantial amounts of protein and carbohydrates. However, they lack minerals and vitamins (micronutrients), phytochemicals, and fibre. In addition to essential nutrients, consumers also demand complete and balanced nutrition for enhancement of their health (Kumar et al., 2023a, 2023b). Selected fruit pomaces act as functional ingredients in several bakery products in fibre and powder forms (Table 5).

**Table 5:** Application of selected fruit pomaces in bakery-based products and their quality characteristics.

Product developed	Country of study	Type of pomace used	Quantity added	Outcomes	Reference
Biscuit	Italy	Apple	10 and 20 %	10 % and 20 % addition of apple pomace in wheat flour reduced glycemic index of biscuit to 65 and 60 correspondingly	Alongi et al. (2019)
	Greece	Apple	32.5, 34, and 49 %	Production of gluten-free biscuits of superior quality with acceptable physical, sensory, and textural properties, containing 32.6 %–47.58 % dried apple pomace	Skaltsi et al. (2022)
	Poland	Grape	10, 20, and 30 %	10 % addition of grape pomace increased total dietary fibre to approximately 88 %; The overall acceptability was best with 10% pomace	Mildner-Szkudlarz et al. (2013)
	China	Grape	0–20 %	Sensory examination of biscuits prepared from 10 % supplementation of grape pomace powder (GPP) displayed good overall acceptability	Lou et al. (2021)
	India	Orange	5–15 %	Increase in fibre content was attributed to increased orange pomace powder content; Sensory analysis of 10 % pomace sample showed better acceptability compared to other samples	Khule et al. (2019)
	Vietnam	Pineapple	10, 20, 30, and 40 %	Pineapple pomace addition increased the antioxidative capacities, total dietary fibre along with hardness of the final product	Nguyen et al. (2024)
	India	Pineapple	5, 10, 15, and 20 %	Amongst the treatment groups, biscuits produced from 15 % pineapple pomace powder received highest sensory scores with panel members (8.27) and ultimate consumers (8.39) having overall acceptability	Maurya and Mogra (2023)
Bread	Poland	Apple	5, 10, and 15 %	Wheat bread made from 5 % whole pomace exhibited the best scores (small baking loss, good volume, low crumb hardness compared to control group on the baking day and during storage)	Gumul et al. (2019)
	Italy	Grape	5, and 10 g/100g	GPP addition increased total phenolic content, total dietary fibre and antioxidant capacity; Sensory evaluation of GPP fortified bread had significant influence on the global flavour, acidity, astringency, and wine smell of the samples with no any impact on the overall acceptability of the bread	Tolve et al. (2021)
	Italy	Grape	5, and 10 g/100g	The GP10 sample showed highest (p < 0.05) phenolic content with 127.76 mg/100 g of dry matter (DM), the next being GP5 with 106.96 mg/100 g of DM; Also, the *in vitro* anthocyanin bioaccessibility increased in GP5 as well as GP10 breads while its movement from gastric to small intestine digestion phase (average value 24%)	Rocchetti et al. (2021)
	Ireland	Orange	0–8 %	Orange pomace (OP) exerted most significant (p < 0.0001) impact on the hardness of bread after 2 h and 24 h of baking. The optimized formulation contained 5.5 % OP and 94.6 % water, with a 49 min proofing time; The total fibre content in OP containing bread was increased to 3.9 % as against 2.1 % in the control bread	O’Shea et al. (2015)
	India	Pineapple	5, 7.5, 10, and 12.5 %	Increase in pineapple pomace powder (PPP) concentration increased the fibre content and addition of 5 % PPP to the bread received more acceptability	Darshini et al. (2021)
	Thailand	Pineapple	5, and 10 %	With the increase in PF levels (p < 0.05), the oil- and water-holding capacity of the composite flour (CPF) was enhanced and bread containing CPF-5 owned more acceptability than CPF-10; However, in terms of specific volume and texture, CPF-5 was similar to control but had three times the total dietary fibre	Chareonthaikij et al. (2016)
Cake	India	Apple	10, 20, and 30 %	Increase in apple pomace content (0 %–30 %), decreased the cake volume (850–620 cc); Cakes with 25% pomace had 14.2 % dietary fibre; the total phenol content reported in apple pomace and wheat flour was 7.16 and 1.19 mg/g respectively although cakes made from 0 % pomace had 2.07 mg/g and 25 % pomace blends had 3.15 mg/g content thus, demonstrating that apple pomace could serve as rich source of dietary fibre and polyphenols	Sudha et al. (2007)
	Bulgaria	Grape	4, 6, 8, and 10 %	Increasing the amount of GPP gradually increased lipid, proteins, ash, fibres, free phenolics, total polyphenol, anthocyanin content and antioxidant capacity and decreased the pH and moisture; Addition of GPP significantly improved the free phenolic content, bioavailability, that is limited in bread wheat thus, retaining the nutritional value without affecting their sensorial and technological attributes	Nakov et al. (2020)
	Turkey	Orange	5, 10, and 15 %	Cakes supplemented with orange pomace powder (OPP) at 5 % concentration exhibited similar hardness values and volume index to the control and also received maximum acceptability from the panellists	Kırbaş et al. (2019)
Cookies	Serbia	Apple	25, 50, and 75 %	The cookies made from coarse, 50 % apple pomace flour (APF) possessed dietary fibre (DF, 21 g/100 g), higher total flavonoids content (TFC), total polyphenolic content (TPC), as well as antioxidant (AO) capacity than control; The prepared cookies retained health promoting compounds, maintained crispy texture and intense fruity aroma	Zlatanović et al. (2019)
	Turkey	Grape	5, 10, and 15 %	Grape pomace (GP) addition to cookie blend had no significant impact on the thickness, width, and spread ratio, however, use of more than 10 % GP flour in cookies, general affordability and acceptability significantly decreased	Acun and Gül (2014)
	India	Grape	5, 10, 15, and 20 %	The increase in colour intensity was observed in wine GPP when its concentration was increased in cookies; the organoleptic testing showed that 5 % wine GPP registered maximum score	Maner et al. (2017)
	India	Grape	15, 20, 25, and 30 %	Blending the cookie mix with 15 % GPP found good acceptability	Sharma et al. (2018)
	India	Pineapple	10, 20, 30, and 40 %	Sample having 30 % PPP contained maximum fibre, best sensory score, physical and nutritional parameters and good organoleptic properties as compared to other samples	Kumar et al. (2020)
	India	Pineapple	5, 10, and 15 %	The incorporation of PPP produced low gluten content cookies displaying improved flour properties, dough properties, and antioxidant activity	Jose et al. (2022)
	Serbia	Tomato	15, and 25 %	The sensory analysis revealed that substitution of 15 % reduced surface roughness, granularity, fracturability, and increased intensity of caramel flavour; Substitution of 25 % caused cookie softening to higher extent as well as more prominent tomato flavour	Tomić et al. (2016)
	India	Tomato	5, 10, 15, 20, and 25 %	Cookies with 25 % tomato pomace inclusion had dietary fibre of 10.23 % and total phenolics of 6.20 mg GAE/100g in contrast to control, thus indicating that pomace could serve as abundant source of dietary fibre and polyphenols.	Bhat et al. (2017)
Crackers	Portugal	Grape	5, 10, and 15 %	Inclusion of 10 % GP received best overall acceptability	Marcos et al. (2023)
	Turkey	Tomato	4, 8, and 12 %	Tomato pomace supplementation led to significant (p < 0.05) enhancement in protein, dietary fibre (insoluble, soluble, total), ash, minerals (Ca, Mg, P, K, Mn, Fe, Zn), total phenol content, and antioxidant capacity	Isik and Topkaya (2016)
	Bulgaria	Tomato	4, 6, 8, and 10 %	Blending 10 % tomato pomace exhibited best sensory parameters and improved physiochemical attributes	Nakov et al. (2022)
Muffins	Pakistan	Apple	5, 10, and 15 %	Supplementation with apple pomace powder (APP) derived from two varieties significantly increased crude fibre, ash, and phenolic content; Highest sensory attributes were showcased with 15% addition and muffins with 10% APP showed better results on hedonic scale	Younas et al. (2015)
	Italy	Grape	15 %	Addition of 15 % GPP irrespective of the particle size produced muffins that were rich in total dietary fibre of >3/100 g and antioxidants, and hence, could be labelled as “source of fibre” based upon the EC Regulation No. 1924/2006	Troilo et al. (2022)
Pasta	Poland	Apple	10, 20, 30, and 50 %	Substitution of apple pomace in pasta blend resulted in enhanced pro-health compound levels such as total polyphenols, quercetin derivatives, phenolic acids, dihydrochalcones, and flavon-3-ols along with dietary fibre and decreased the maximum cutting energy and hardness of the supplemented pasta compared to the control pasta	Gumul et al. (2023)
	Italy	Grape	5, and 10 %	Nutritionally, increasing the amount of GP resulted in significant depletion in rapidly digestible starch along with increment in slowly digestible starch concentration *in vitro*, with reduction (p < 0.05) in forecasted glycemic index	Tolve et al. (2020)
	Romania	Grape	3, 6, and 9 %	Incorporation of 6 % pomace led to improvement in functional and sensory properties	Gaita et al. (2020)
	India	Pineapple	5, 10, 15, 20, and 25 %	Supplementation of 5 %–25 % pineapple pomace, led to improved tolerance index	Munjaji et al. (2022)
Rock bun	Ghana	Pineapple	5 %	Best sensory acceptance	Badjona et al. (2019)
Sugar-free bars	Poland	Grape and apple	10 and 20 g	Fortification with these pomaces significantly increased the moisture content, soluble fibre content, and decreased the levels of antioxidants found in them; Strength of supplemented cereal bars was also increased	Blicharz-Kania et al. (2023)

Gluten-free bread prepared from 5 % apple pomace inclusion showed highest organoleptic scores along with high phenolic content (Gumul et al., 2021). The total phenolic content was 2.5 times higher than control, and the flavonoid, phloridzin, and phenolic acid quantities were 8, 21, and 4 times greater in this bread compared to control. Rainero et al. (2022) have designed fortified breadsticks by substituting wheat flour with different grape pomace powder (GPP) concentrations (0, 5 and 10 g/100 g). Inclusion of GPP affected the rheological attributes of the stick doughs by enhancing water absorption as well as tenacity (*P*) simultaneously. It also reduced the extensibility (*L*), with significantly increased *P/L* value, decreased deformation energy (*W*) and swelling index (G) value. Textural characteristics were also affected by addition of GPP to breadstick dough, displaying reduced fracturability and hardness on increasing the GPP amount in the formulation and evidenced good sensory acceptability. Kruczek and team (2023) have developed apple pomace (AP) supplemented gluten-free cookies in 15, 30, 45, and 60 percent proportions. It was observed that AP in the cookies led to increase in quercetin derivative, phenolic acid, dihydrochalcone, and flavan-3-ol content. Elevated levels of protein, minerals, and fat was also observed. Increased apple pomace share brought significant increment in total fibre, insoluble and soluble fraction content, but also resulted in enhanced darkening and hardness of the cookies, with reduced volume. Pinot Grigio white wine grape pomace (WWGP) and Pinot Noir red wine grape pomace (RWGP) replaced wheat flour at different concentration for bread (5, 10, and 15 %), RWGP for brownies (10, 15, 20, and 25 %), and RWGP for muffins (5, 10, and 15 %) or WWGP for muffins (10, 15, and 20 %) were utilized by Walker et al. (2014). The finished food products were assessed for total dietary fibre (DF), radical scavenging activity (RSA), and total phenolic content (TPC). The results demonstrated that 15 % RWGP supplemented muffins and bread samples had highest RSA (80.70 and 1526 mg AAE/serving for bread and muffins respectively) and TPC values (68.32 mg and 2164 mg GAE/serving for bread and muffins respectively). 10 % RWGP fortified brownies had highest RSA value of 115.52 mg AAE (ascorbic acid equivalents)/serving as compared to the control having highest TPC value of 1152 mg GAE/serving. 15% RWGP supplemented breads and muffins and 25 % RWGP brownies showcased highest DF content of 6.33 g, 12.32 g, and 7.73 g/serving, respectively.

Devi and researchers (2023) included dietary fibre from pineapple pomace (PP) in soy flour and semolina blends for the development of protein-fibre rich pasta. The colour, sensory attributes, and texture of the prepared pasta were studied. Hedonic scale was used to carry out sensory analysis and the results showed that pasta sample fortified with 3 % dietary fibre and 5 % soy flour displayed best attributes. Gerardi and team (2023) utilized GP for fortification of pasta. In case of uncooked GP fortified pasta, the bound and soluble phenolic content increased significantly. The cooked pasta contained double amount of soluble phenols and no bound phenols. The pasta prepared from whole grape pomace flour (WGPF) fortification doubled the fibre component, isoprenoids (carotenoids and toco-chromanols), and enhanced soluble polyphenols by 10 times, while retaining the fatty acid (unaltered) content after cooking. Corresponding to the polyphenol content, the antioxidant activity was also higher than the control pasta. The fortified pasta (cooked and uncooked) was analyzed for volatile compounds and the results indicated improved aromatic profile in comparison to the control batch.

### 5.2 Milk-based foods

Fruit pomace powders have been used in various dairy products *viz*., curd, yogurt, chocolate, milk beverages, and semi-hard cheeses (Table 6). Popescu et al. (2022) have developed yoghurt with AP powder addition in 0.2–1.0 % proportion and witnessed significant increment in antioxidant capacity, which could be correlated to the pomace content. The textural properties were reported to have improved, when the yogurt was stored for 20 days and hence, led to significant syneresis reduction. The sensory analysis and textural characteristics of the preparation during storage for 1–20 days displayed that AP samples in 0.6–0.8 % proportions evidenced the highest score. A study by Marchiani et al., 2016a, Marchiani et al., 2016b utilized GP from Moscato, Pinot, and Chardonnay noir varieties adding polyphenolic compounds to the formulation during storage (3 weeks). Results have revealed that yogurt enriched with grape skin flour offered significantly higher TPC (+55 %), acidity (+25 %), and antioxidant activity (+80 %) and lower fat (−20 %), syneresis (−10 %), and pH than control. Vanillic acids and procyanidin B1 were identified in yogurt containing Pinot noir flour whereas phenolic compounds such as gallic acid, quercetin, and catechin were detected in yogurts containing Chardonnay or Moscato grape skins. Another study utilized orange pomace powder for supplementing yoghurt in three proportions (1, 2, and 3 %) (Acharjee et al., 2021). Increasing the concentration of the powder and storage time led to increased acidity of the yoghurt. Additionally, the syneresis of supplemented samples was reported to be lesser than the control, at the same increased with higher levels of enrichment. Sensory analysis indicated that the 1 % enriched yoghurt received acceptability amongst the panellists.

**Table 6:** Application of selected fruit pomaces in milk-based products and their quality characteristics.

Product developed	Country of study	Type of pomace used	Quantity added	Outcomes	Reference
Yoghurt	India	Apple	2.5, 5, 7.5, and 10 %	Fibre inclusion in yoghurt led to decreased fat content (1.65–1.59 %) and acidity (0.15–0.09 %) with increase in concentration of fibre; Sensory analysis showed that yoghurt supplemented with 5 % pomace was judged the best among all and hence optimized to prepare fibre-rich acidophilus yoghurt possessing desirable sensory and quality attributes	Issar et al. (2017)
Yoghurt	Serbia	Apple	1, 3, and 5 %	The highest firmness, viscosity index values, cohesiveness, and colour and taste scores were obtained from 3 % APF supplemented yogurt, thus indicating 3 % APF as the optimal amount for producing novel yogurt with desirable functional properties	Jovanović et al. (2020)
Yoghurt	United States of America	Grape	1, 2 and 3 %	GP addition resulted in peroxide value reduction up to 35–65 %, wherein 1% GP inclusion was most liked by consumers	Tseng and Zhao (2013)
Yoghurt	India	Grape	0.5, 1, and 1.5 %	GP of 1 % displayed highest sensory score	Subhashini et al. (2018)
Yoghurt	India	Pineapple	0.1, 0.25, and 0.5 %	The texture parameters, consistency, cohesiveness, and firmness showed significant variation (p < 0.05) during the storage period, thus demonstrating reinforcement of structure of yogurt; Also, significant variations in syneresis, titratable acidity, and pH were observed in yogurt supplemented with PP in comparison to control; The sensory analysis showed good acceptability in 0.1, and 0.25 % PP concentrations compared to 0.5 %	Meena et al. (2022)

Lassi, a traditional Indian beverage, prepared from yoghurt is consumed in summer season as a refreshing drink. Kumar et al. (2023) have developed a tomato pomace blended lassi with varying rates of supplementation (0.5 %, 1 %, 1.5 %, and 2 % w/v) of milk and was added while heating the milk. Lassi prepared from 1 % tomato pomace enrichment showed better sensory score, physico-chemical properties and received overall acceptability of 7.5. Additionally, 1 % tomato pomace blended lassi had 2.67 % protein, 2.53 % fat, 0.66 % ash, 18.81 % total solids, 12.37 mg GAE/100 g and 21.81 % DPPH (2,2-Diphenyl-1-picrylhydrazyl) inhibitory activity. The lassi prepared from the pomace powder was reported to be stable till twelve days compared to the control samples that turned unacceptable after nine days of storage. Dahi (curd) has been prepared by utilizing freeze-dried pineapple pomace powder (FPP) in a range of 0.5–2.5 g/100 ml (Gumber et al., 2024). The prepared dahi was further investigated for their storage study and sensory evaluation. The sensory analysis results revealed that dahi containing 1 % FPP received significant organoleptic acceptance. Powders derived from three GP of varieties Barbera and Chardonnay before and after distillation were supplemented at 0.8 % w/w and 1.6 % w/w concentration levels into hard (Cheddar) and semi-hard cheeses (Italian Toma-like) in order to increase polyphenolic content (Marchiani et al., 2016a, Marchiani et al., 2016b). The results showcased those cheeses fortified with GP from Chardonnay variety after distillation showed highest RSA and TPC values at ripening completion (30 days for Italian Toma-like and 120 days for Cheddar cheeses). A study on dried grape pomace (DGP) was conducted and it was used in compound chocolate (CC) as bulking agent to partly substitute sugar, whey powder, and milk powder (Bursa et al., 2022). The outcomes explain that DGP was reported to be suitable for use as bulking agent in CC by partially substituting milk powder, sucrose, and whey powder and increased the functional properties as well as decreased the CC cost. For obtaining satisfactory levels of RSV and TPC, and best acceptable rheological attributes the optimal DGP usage levels were identified (7.1 %–10.0 %).

### 5.3 Meat-based foods

Dried apple pomace, in 7 and 14 % proportion, was included in pork meat for preparation of Italian salami, and was then subjected to ripening for 25 days (Grispoldi et al., 2022). The results revealed slightly higher overall acceptability for 7 % AP in comparison to 14 % AP containing samples, and it was observed that replacement of pork with AP allowed production of Italian salami having sensory properties similar to those achieved with classic recipes. The fortified salami had better phenol and fibre content, lower fat content and calories, representing interesting characteristics. Goshtaba is a popular emulsion-based meat dish of Kashmiri wazwan prepared from comminution (ground) of meat product (Samoon and Sharma, 1991). In another study by Rather et al. (2015), investigations on apple pomace powder (APP) were carried out to evaluate its role in replacing fat at different concentrations (1–5 %) in goshtaba formulations with low fat and were subjected to textural, physico-chemical and sensory analysis (Rather et al., 2015). The T0 (apple pomace 0 % + 20 % fat) goshtaba samples was found to be significantly higher in cohesiveness, hardness, chewiness, and gumminess, and had lower springiness as compared to low fat formulations with or without APP (p < 0.05). Sensory analysis has indicated that goshtaba formulations with reduced fat content (20–10 %) and supplementation with APP (1 and 3 %) showed overall palatability similar to T0 (20 %) goshtaba. Supplementation of white grape pomace to pork burgers at different proportions (0.5, 1, and 3% w/w) was done to improve preservation and was compared to burgers containing sulfites and control (without pomace or sulfites) (Martín-Mateos et al., 2023). Pomace limited protein and lipid oxidation development on storage (p > 0.05), whereas sulfites showed no effect. Hence, the usage of white wine pomace in burgers could potentiate antioxidant effect with limited colour-protective or antimicrobial effect for preserving pork burgers. Another study has been designed on silver carp fish for determining the antioxidant effect and phenolic content in grape pomace extract supplemented fillets during refrigerated storage (Hasani and Alizadeh, 2015). The fillets were treated with grape pomace extract at various proportions (0, 2, and 4 %, g extract*/*100 g flesh) and refrigerated (4 °C) for 15 days. The findings revealed that the pH, thiobarbituric acid concentration, peroxide value was increased while a decrease in the iron levels was noticed in all treatment groups during storage (p < 0.05). The quality parameters influenced the phenolic contents in treated groups as compared to control. Additionally, grape pomace extracts also delayed lipid oxidation considerably in the fillets during refrigerated storage.

Fresh and steamed pineapple pomace (SPP) prepared under pressure were utilized for production of dietary fibre concentrates (DFCs) as well as evaluation of its effects on mixing with meats on Vienna-type sausage characteristics (Montalvo-González et al., 2018). On applying cubic model equations on the data, it was found that increasing the SPDFC levels in ternary mixture led to reduced effect on moisture, nitrites, shrinkage and shear force in sausages, whereas an increase in antioxidant polyphenols and carotenoids was noticed. The study illustrated that steamed and hot-air-dried (SPDFC-HD) was designed and produced to exhibit characteristics for use as ingredient in functional sausages. Skwarek and Karwowska (2022) have assessed dry fermented sausages for the effect of tomato pomace (TP) on their physicochemical characteristics, fatty acid profile and antioxidant potential having reduced nitrite content. Four different formulations of sausage were prepared by varying the concentration of freeze-dried pomace (0.5, 1 and 1.5 %, control sample). Results have showed that inclusion of freeze-dried pomace increased the antioxidant activity proportionate to the increase in TP concentration, attributed to high lycopene content and strong antioxidant property of tomato. Additionally, TP exhibited antimicrobial properties in raw fermented samples of sausages as revealed by low number of *Enterobacteriaceae* in samples with higher concentration of pomace. Also, sausages with lower nitrite levels and added TP evidenced higher redness, probably having a positive influence on the consumer assessment. The sausages with 1.5 % TP showed most promising results amongst all the groups. Table 7 summarizes enrichment applications of diverse meat-based food items utilizing selected fruit pomaces.

**Table 7:** Application of selected fruit pomaces in meat-based products and their quality characteristics.

Product developed	Country of study	Type of pomace used	Quantity added	Outcomes	Reference
Beef burger	Italy	Apple	4, and 8 %	The results concerning sensory and colour analysis of the supplemented products were graded better than control, with improved phenol and fibre content, the neutral flavour, symbolizes the most fascinating characteristics of pomace fortified burgers	Pollini et al. (2022)
Beef hamburger	Egypt	Grape	2, and 4 %	GP inclusion brought about significant impact on pH at the very beginning (zero time), wherein the pH values of 2 %, 4 %, and control samples was 6.41, 6.22, and 6.68 respectively; Significant influence on thiobarbituric acid (TBA) was evidenced at 4th week of study, with results showcasing increased microbial growth owing to storage of the product and the GP addendum in beef burger led to reduction of total bacterial count as compared to control batch	Abdelhakam et al. (2019)
Low-fat beef burger	Egypt	Tomato	12.5, 25, 37.5, and 50 %	Beef burger prepared with tomato fibre pectin (TFP) derived from tomato pomace as fat replacer reduced cooking loss, and decrease in burger diameter reduction; Also, samples having 12.5 and 25 % TFP received highest acceptance	Namir et al. (2015)
Buffalo meat patties	India	Apple	2, 4, 6, and 8 %	Increase in APP incorporation contributed to increased cooking yield, water holding capacity, meat emulsion stability; The sensory analysis revealed that 6 % incorporation of APP was acceptable	Younis and Ahmad (2018)
Chicken patties	Spain	Grape	30 and 60 mg/kg	Pomace concentrate addition caused significant inhibition of lipid oxidation of cooked and raw breast chicken patties on comparison with bird fed control diet samples after 20 days and 6-month frozen storage	Sáyago-Ayerdi et al. (2009)
Chevon meat patties	India	Pineapple	2, 4, and 6 %	Inclusion of fibre diminished moisture content and enhanced the fat, ash, and protein content; Addition of 4 % PPP displayed good sensory score	Sunita et al. (2015)
Reduced-fat chicken sausages	Korea	Apple	1, and 2 %	Inclusion of APF in the preparation successfully reduced fat content in emulsion-type sausages, wherein improving the quality characteristics compared to regular-fat control (30 % fat)	Choi et al. (2016)
Chicken sausage	India	Apple and tomato	3, 6, and 9 %	Incorporation of 6 % dry tomato pomace (DTP) and dry apple pomace (DAP) each to chicken sausages led to very good acceptability, storability (15 days of refrigeration), and higher dietary fibre	Yadav et al. (2016)

### 5.4 Other food products

A study by Rehal and Kaur (2023) evaluated the effect of TPP inclusion in Bhujia, an Indian snack at varying levels (0–12.5 %). Results have showed that TPP supplementation up to 5 % imparted high acceptability of the product in terms of sensorial properties. The product yields were also increased compared to control sample without any tomato pomace powder addition. The compositional profiling of the product revealed it to be an abundant source of fibre (3.40 %), fat (20.8 %), protein (16.52 %), antioxidant activity (38.5 %), and energy value (485.32 kcal/100g). The same research group has also worked on preparation of gluten-free ready-to-cook snack by utilizing potato flour and finger millet (50:50) along with tomato pomace (Rehal et al., 2022). The TPP addition had a distinctive influence on the colour and hardness of the product. Moreover, its addition resulted in significant drop in the cooking oil quantity used, frying time, and cooking loss. The snack formulation supplemented with 10 % TPP had high acceptance and was established as the most optimal one. Value-added ketchup has been developed from TPP and compared to ketchup previously prepared from fresh TP (Belović et al., 2018). The comparison revealed that total dietary fibre level of TPP ketchup was greater than fresh TP ketchup pertaining to production process wherein seeds were not removed. Furthermore, the TPP ketchup showed improvement in thermal stability, thus suggesting its possible application in bakery filling. Previtera et al. (2016) have reported that supplementation of lyophilized TPP enhance puree properties. Another study was conducted on pineapple pomace for its maximal utilization in peanut bar formulation using jaggery or cane sugar (Molla et al., 2021). The findings revealed that the prepared peanut bar evidenced abundance of crude protein (13.06 ± 0.05 %), crude fibre (6.48 ± 0.48 %), β-carotene (16.32 ± 0.03 μg/100 g), and vitamin-C (23.28 ± 0.21 mg/100 g) than the marketed sample. However, the physicochemical and nutritional properties of both the nut bar and marketed sample (Badam topi in this case) gradually diminished with increased storage periods.

### 5.5 Edible films/coatings

By-products derived from food are generally composed of proteins, lipids, and polysaccharides, which tend to produce biodegradable packaging, that can be used in combination or separately, and are usually decomposed into water and carbon dioxide. Hence, their application in producing biodegradable packaging can be seen as a potential alternative, generating more sustainable options for preservation of food (Jorge et al., 2023). More recently, the focus has been on the use of combination of diverse types of wastes and by-products from vegetables, fruits, and cereals in producing biodegradable films/coatings (Jorge et al., 2023; Kumar et al., 2023a). A latest development in edible coatings/intelligent functional films has led to their enrichment with bioactive compounds that are derived from fruit pomace extracts.

Teleky et al. (2022) have developed water-soluble and biodegradable packaging materials by using pectin and poly(vinyl alcohol) (PVA). The phenolic or organic extracts of apple pomace (by-products) were used to enrich the produced film-forming solutions. The resultant biofilms presented reduced water solubility and lower water vapor permeability. Enrichment with phenolic compounds facilitated development of resistance against extrinsic and intrinsic factors, thereby opening new avenues for their application in food industry. Biodegradable cassava or tapioca starch film prepared by extruding different AP by-product percentages was evaluated (Carpes et al., 2021). A high total phenolic content of 3.32 mg GAE/g and antioxidant capacity of 2.78 mmol Trolox/g was observed in film having 8 % AP compared to the control showing total phenolic content of 0.71 mg GAE/g and antioxidant capacity of 1.03 mmol Trolox/g. Further testing for its minimum inhibitory concentration (MIC), it was found that films containing AP showed >12.5 mg/mL MIC against *Salmonella thyphimurium*, *Staphylococcus aureus*, and *Escherichia coli*. Gubitosa and team (2023) have reported that grape pomace derived aqueous polyphenolic extract enhanced the antioxidant properties as well as bestowed UV-light screening in sodium-alginate-derived films. Composite film containing sodium caseinate (NaCas), lipidic fraction of tomato pomace (LFTP), and glycerol (25 wt%), at different proportions (0–40 wt%) were prepared and characterized for their antioxidant activities, thermal stability, hydrodynamic, mechanical, and optical properties (Aloui et al., 2019). Results revealed that on increasing LFTP concentration above 20 wt% enhanced the film flexibility in a range of 17–25 %. Besides, LFTP incorporation at 40 % (highest content) also improved thermal stability and decreased absorption of water by N72 % in the NaCas films. In addition, increase in LFTP content caused a significant reduction in light transmission by the NaCas/LFTP composite films. However, the antioxidant activity of the composite films was improved by increasing the LFTP content attributed mainly to the higher total phenolic content of LFTP.

## 6 Enrichment of selected fruit pomaces in animal feeds

The ever-increasing human population, together with the exigent requirement to meet up the rising demand for meat, egg, and milk, has placed the responsibility on farmers and food nutritionists to identify alternative ingredients that could achieve cost-effective feed formulation (Erinle and Adewole, 2022). Among all sectors of agriculture, livestock is the highest growing subsectors in developing nations (Kumar et al., 2023b). Additionally, the public requisition for antibiotic free livestock products has necessitated crucial search for antibiotic substitutes in livestock production (Erinle and Adewole, 2022). Consequently, reducing or removing animal products and substituting them with affordable plant products in functional feed formulations become necessary (Kumar et al., 2023b). Plants sources and/or their by-products such as fruit pomaces offer advantage in being pocket-friendly and hence, has gained much interest. Fascinatingly, fruit pomaces consist of appreciable protein, dietary fibre, and phenolic compounds, and hence, their adoption could benefit the livestock industry in dual capacity that includes their use as antibiotic substitutes and conventional feedstuff alternatives (Erinle and Adewole, 2022). The upcoming sub-sections cover detailed description on selected fruit pomaces finding enrichment applications in diverse animal feeds.

### 6.1 Chicken

A study was conducted on healthy broiler chicks (Ross 308) that were 480 days old, with mixed sex and were fed on dried apple pomace (Aghili et al., 2019). The dried apple pomace was used at incremental levels (4 %, 8 %, 12 %, 16 %, and 20 %) in broilers diet (with enzyme and without enzyme). The findings revealed that the gradual increments of dried AP deteriorated growth performance, modified total antioxidant capacity, antibody titer production, and blood profile of broilers. Haščík et al. (2023) have also determined Alibernet variety derived Alibernet red grape pomace (ARGP) effects on inclusion into Ross 308 broiler chicken diet containing fatty acid (FA) and essential amino acid (AA) in the thigh and breast meat. Three experimental groups based on the varying amounts of ARGP in feed were assigned, first (E1) being 1 % ARGP/kg enriched feed mixture (FM), second (E2) being 2 % ARGP/kg enriched FM and third (E3) being 3 % ARGP/kg enriched FM. The investigations on breast muscle revealed no significant impact on the AA profile, with leucine and lysine being the most prevalent AAs. In case of thigh muscle, significant differences were observed in the valine, threonine, methionine, histidine, and cysteine content in males. The FAs profiling stated that ARGP inclusion influenced monounsaturated FA, oleic acid in the breast muscle (with no gender disparity), with significantly high content (p ≤ 0.05) across all experimental groups; E1 containing 36.05 g/100 g, E2 containing 35.60 g/100 g and E3 containing 36.79 g/100 g as compared to the control group containing 31.88 g/100 g. Another study by Romero et al. (2022) has documented that supplementation with grape pomace in eggs at 60 g/kg enhanced the polyunsaturated FA proportion in the yolk along with improvement in its lipid oxidation stability during storage of eggs, while no effect was observed with grape extract. Assessment of grape products on hen performance revealed reduction of feed intake, average egg weight, and feed conversion ratio but had no effect on the daily egg mass and production. In conclusion, it was implied that inclusion of grape products in laying hen diet enhanced the quality of egg but reduced the egg weight and feed intake. Hu and researchers (2023) have reported that blending 3 % PP in broiler chicken (Ross 308) diet had no harmful effects on the meat quality or growth performance.

Gungor and team (2024) have investigated *Aspergillus niger*-fermented tomato pomace (FTP) and tomato pomace (TP) for their effects on the antioxidant status, some carcass traits, growth performance and intestinal microflora in one-day-old Ross 308 male chicks. The findings showcased that presence of TP in diet increased SOD and serum glutathione peroxidase (GPx) but had no effect on the growth performance. Inclusion of FTP in diet raised serum SOD and GPx and improved feed conversion ratio. Carcass traits, pH, malondialdehyde level, breast meat colour and cecal microflora had not been affected by the dietary treatments. A study by Zadeh and group (2023) analyzed dietary supplementation of L-arginine (L-Arg) and TP at different proportions on the reproductive performance, semen biochemical components, and sperm characteristics of aged (58 weeks old) commercial Ross 308 male broiler. Dietary treatment groups were assigned as control, 5 % TP, 10 % TP, 15 % TP, and above 10 % of recommended L-Arg supplementation (control group (CON), tomato pomace supplemented (TPS)-5, TPS-10, TPS-15, and LAS-10, respectively). The results showed that the TPS-15 group increased the semen volume in comparison to the L-arginine supplemented (LAS)-10 group and CON group (on 9 weeks), throughout the course of study (p < 0.05). A significant increase in the sperm concentration in TPS-10 as well as TPS-15 groups compared to other experimental groups was observed. Additionally, the fertility rate and sperm penetration were increased in both the TPS-10 group and TPS-15 group compared to LAS-10 and CON groups (p < 0.05). On the other hand, a reduction in hatchability was noticed in LAS-10 group. The study concluded that 15 % TPS in diet is recommended for improvement in reproductive performance of commercial broiler (aged male) breeders.

### 6.2 Fish

Fried tilapia (*Oreochromis niloticus*) fish fed on apple pomace protein enriched diet presented increased body mass (up to 44 %), thereby demonstrating the use of AP biotransformed as a brilliant food supplement (Vendruscolo et al., 2009). Pulgar et al. (2021) have evaluated the outcome of micro-encapsulated grape pomace extract (MGPE) derived dietary polyphenol supplementation in diet. The MGPE was obtained from the residual by-product of wine industry in a soybean rich diet impacting the growth performance, gut microbial communities, and plasma antioxidant capacity of rainbow trout fish (*Oncorhynchus mykiss*) monitored at intervals (30 days, 60 days, and 90 days). Results have shown that fish fed on MGPE supplementation illustrated increased growth and high antioxidant activity. Correspondingly, a microbiome analysis of the fish gut displayed a time-dependent fall in microbial richness along with diet-dependent alterations in bacterial microbiota after a 60-day treatment. The data also indicated that the cooperative interaction network among gut bacteria could contribute to the adjustment, potentially explaining the improvement in antioxidant capacity and growth observed in fish fed on a diet with high soybean meal (SBM) supplemented with MGPE. Kesbiç and researchers (2022) have evaluated by-product extract of tomato paste impact on dietary supplementation in common carp fish (*Cyprinus carpio*). On completion of the feeding trial, it was found that fish fed on experimental diets displayed improved growth performance, a vital component of aquaculture, along with a diminished feed conversion ratio, an additional critical parameter. Hematological parameters such as hemoglobin content (Hb), hematocrit (Hct), and erythrocyte count (RBC) were increased in fish receiving enriched diet. Also, cholesterol, triglyceride, and blood glucose levels in the carp fed on tomato paste supplemented diets were reduced while albumin, globulin, and total protein levels were increased. A study by Abedalhammed et al. (2020) has showed the effect of addition of dried TP at different levels (0 for T_1_, 5 % for T_2_, 10 % for T_3_, and 15 % for T_4_) to feed for its productive performance in the common carp (*Cyprinus carpio* L.). The experimentation results displayed the prospects for using TP in 15 % proportions in common carp fish diets and showed significant improvement in the white blood cell and red blood cell count compared to other treatments.

### 6.3 Pig

Weaned pigs were utilized for investigating the effect of fermented AP on plasma antioxidant and biochemical indicators as well as the fecal microbiota (Ao et al., 2022). The AP group witnessed increased concentration of superoxide dismutase and albumin, while decreased levels of malondialdehyde and aspartate aminotransferase were noticed. It is fascinating that the *Lactobacillus* abundance and relative abundance of the pathways involving genetic information processing, evidenced significant increase in AP group. Ajila and team (2015) have documented that fermented AP at 5 % proportion in pig diet showcased 36 % increase in protein content. The feed conversion efficiency in the experiment group increased to 92.1 ± 3.6 starting from a baseline efficiency of 55.4 ± 4.5 during the feed experiment. Recently, Tian et al. (2023) have examined GP for its effect on inflammation, meat quality, growth performance, and gut barrier function in finishing pigs. The results showed that the treatment samples displayed a significant reduction in water loss, reactive oxygen species (ROS), IL-1β, MDA content, and diamine oxidase (DAO) (p < 0.05). IgA, IgM, IgG, total antioxidant capacity (T-AOC), CAT, interferon-gamma (IFN-γ), and SOD showed significant increase on comparison to control samples (p < 0.05). In the meantime, a significant difference (p < 0.05) in mRNA expression of occludin (a tight junction protein) compared between treatment and control groups was observed. The metagenomic sequencing analysis indicated that GP caused significant decrease in relative abundance of *Streptococcus* and *Treponema* (p < 0.05). Kafantaris et al. (2018b) have studied GP use as feed additive for weaned piglet diet in an attempt to develop novel feedstuffs and investigate their possible beneficial effects on productivity, welfare, and meat quality. Results revealed that piglets feeding on GP supplemented diet significantly augmented antioxidant mechanisms invariably in almost all tissues, as depicted by enhancement in hydrogen peroxide (H_2_O_2_) decomposition activity, total antioxidant capacity (TAC), and reduced glutathione (GSH) compared to control group. On checking the FA composition of meat, it was found that piglets on GP blended diet significantly increased the n-3 fatty acids while significantly decreasing the n-6/n-3 ratio as compared to control (p < 0.05). A study by Correia et al. (2017) assessed TP for its nutritional effects on the muscle *(longissimus lumborum)*, liver, and subcutaneous fat in young pigs. The pomace was blended with two different fat sources (soya bean oil or lard). 4-week-old 40 male pigs were randomly divided into 4 dietary treatment groups by using two fat sources (soya bean oil or lard) and two fibre sources (5.0 % tomato pomace or wheat bran) for a period of 5-weeks. The presence of TP showed no improvement in the fatty acid profile, colour, lipid oxidation or cholesterol content, with no evidence of detectable levels of β-carotene or lycopene in the meat (p > 0.05). But a positive increment in the a-tocopherol content of liver and meat was noticed in TP (p < 0.05) fed pigs. The soya bean oil in comparison to lard increased the polyunsaturated fatty acids (PUFA) and monounsaturated fatty acids (MUFA) proportions along with and decrease in saturated fatty acids (SFA) percentages (p < 0.05).

### 6.4 Grazing animals

Recently, Gadulrab et al. (2023) have studied the impact of dried apple pomace fed dairy cattle on fatty acid concentration, ruminal fermentation, methane production, and microbial populations. The experiment was conducted on four commercial, rumen-cannulated dairy cows and lasted for 64 days. The control animals were fed on a standard diet, whereas the experimental animals received a standard diet along with 150 g/kg DM (dry matter) supplemented dried apple pomace. The results revealed a change in ruminal fermentation variable number, owing to inclusion of dried AP to standard diet. The ruminal pH showed a slight increase (p < 0.01) while a decrease (p < 0.01) in ammonia concentration by 46 % was noticed. The total protozoa count witnessed a significant decrease (p < 0.01) while total volatile FA showed an increase (p < 0.01). Additionally, 8% reduction (p = 0.05) in methane emission was credited to dried AP feeding. The study illustrated that dietary dried AP exerted a positive effect (150 g/kg DM) on the ruminal metabolism such as decrease in methane emissions and ammonia concentration, along with increase in milk production, higher nutrient digestibility, and total volatile fatty acids (VFAs) in ruminant cattle. Arend et al. (2022) have recently evaluated high amount of grape pomace (HGP) effects on dietary supplementation to finishing cattle on product quality, FA composition, and carcass traits of beef. Holstein × Jersey crosses (n = 24) received two of diets: Control (CON) composed of typical finishing diet and a 58 % GP containing finishing diet. FA profile and sensory analysis was conducted by collecting *Semimembranosus* (SM) and *Longissimus lumborum* (LL) muscle from each carcass. Comparison between the HGP diet to CON revealed reduction in malondialdehyde (MDA) concentration and lipid oxidation of SM and LL steaks over time. Total PUFA and conjugated linoleic acid (CLA) levels were higher (p ≤ 0.02) for HGP fed steers in comparison to the CON group. Abdollahzadeh and researchers (2010) have evaluated ensiled mixed tomato and apple pomace (EMTAP) addition instead of alfalfa hay to Holstein dairy cow diet impacting their performance. The animals received a mixture of alfalfa hay along with EMTAP at three different levels (0 %, 15 %, 30 %) for a duration of 63 days. The findings showed that milk composition percentage had no significant difference between the diets but significant (p < 0.05) differences in feed efficiency (FE), some nutrient digestibility, and milk production were observed between diets. In conclusion, it was found that concurrent use of tomato pomace and apple pomace (in 50:50 ratios) improved the nutritional value and EMTAP substitution up to 30 % was efficient in diet without showing any harmful effect on dairy cow's performance.

Bennato and researchers (2023) have analyzed the effects of GP supplementation in lambs’ diets. Two groups; control group (CTR) and experimental group (GP+) were fed on a standard diet and with a 10 % GP diet based upon DM respectively for 45 days. On analysis of meat samples, no significant differences were observed in cooking loss, drip loss, total lipid amount, and meat colour among the two groups. On the other hand, the feeding strategy in GP + group influenced the fatty acid composition of meat, wherein increase in relative percentages of vaccenic, rumenic, and stearic acids was observed. The cooked meat samples on 5-day storage at 4 °C showed an increase in 1-octen-3-ol and nonanal owing to GP supplementation in diet and a significant decrease in hexanal levels, an oxidation indicator; which enhanced resistance towards oxidation in GP supplemented samples and was further confirmed by testing thiobarbituric acid reactive species (TBARS). Kafantaris et al. (2018a) have reported that GP fed lamb shows significant increase in the long chain n-3 FAs, docosahexaenoic acid, and eicosapentaenoic acid content and reduced the ratio of n-6/n-3 in the meat in comparison to control group.

## 7 Conclusion

The valorization of fruit pomaces into functional food and feed has the potential to reduce environmental pollution and enhance human well-being by promoting physical vigor to fight against various diseases. The thorough examination of the potential of bioactive elements in fruit pomaces on persons with cancer, poor immune, and other illnesses is still pending, despite the promising results observed in a few fruit pomaces, such as in the treatment of bowel syndrome and diabetes. Due to a dearth of literature availability, it is important to note that this review does not address all fruit pomace types. Additional research is necessary to explore the potential of utilizing different fruit pomaces for the creation of functional food or natural preservatives for animal feed and food applications. This research aims to enhance certain nutritional characteristics while maintaining sensory appeal.

## CRediT authorship contribution statement

Harsh Kumar: Writing–initial and Final draft. Shivani Guleria: Literature collection, Editing. Neetika Kimta: Literature collection, Editing. Eugenie Nipovimova: Conceptualization, Visualization. Rajni Dhalaria: Literature collection, Editing. Daljeet Singh Dhanjal: Figures designing, Editing. Nidhi Sethi: Conceptualization, Literature collection, Editing. Suliman Y Alomar: Conceptualization, Editing. Kamil Kuča: Conceptualization, Visualization, Supervision.

## Declaration of competing interest

The authors declare that they have no known competing financial interests or personal relationships that could have appeared to influence the work reported in this paper.

## Data Availability

Data will be made available on request.
